# Robotic-assisted left atrial appendage occlusion: an important complementary option in the surgical management of atrial fibrillation

**DOI:** 10.1093/icvts/ivaf082

**Published:** 2025-04-07

**Authors:** Alfonso Agnino, Laura Giroletti, Eduardo Celentano, Ascanio Graniero, Ernesto Cristiano, Matteo Parrinello, Giovanni Albano, Mario Gasparri, Stefano Schena

**Affiliations:** Department of Cardiovascular Surgery, Humanitas Gavazzeni-Castelli, Bergamo, Italy; Department of Cardiovascular Surgery, Humanitas Gavazzeni-Castelli, Bergamo, Italy; Department of Cardiac Electrophysiology, Humanitas Gavazzeni-Castelli, Bergamo, Italy; Department of Cardiovascular Surgery, Humanitas Gavazzeni-Castelli, Bergamo, Italy; Department of Cardiac Electrophysiology, Humanitas Gavazzeni-Castelli, Bergamo, Italy; Department of Cardiac Anesthesia, Humanitas Gavazzeni-Castelli, Bergamo, Italy; Department of Cardiac Anesthesia, Humanitas Gavazzeni-Castelli, Bergamo, Italy; Division of Cardiothoracic Surgery, Medical College of Wisconsin, Milwaukee, WI, USA; Division of Cardiothoracic Surgery, Medical College of Wisconsin, Milwaukee, WI, USA

**Keywords:** robotic cardiac surgery, left appendage closure, atrial fibrillation

## Abstract

**OBJECTIVES:**

The growing popularity of minimally invasive treatment of atrial fibrillation (AF) has shown increasing interest in concomitant left atrial appendage occlusion (LAAO). Surgical robotic technology adds advantages such as magnified visualization, enhanced dexterity of movement and decreased invasiveness. The aim of this study is to evaluate the effectiveness and early outcomes of robotic-assisted LAAO.

**METHODS:**

This is an observational, multicentre, retrospective study of patients with AF who underwent robotic-assisted LAAO. In-hospital mortality, perioperative complications, length of stay (LOS) and imaging-driven (cardiac CT scan, transesophageal echocardiography [TEE]) efficacy at 3-month follow-up were analysed.

**RESULTS:**

Between August 2019 and June 2024, 194 patients with documented AF (70.4% male, mean age 67.7 ± 10.1) underwent robotic-assisted epicardial LAAO. The procedure was performed under TEE guidance in 193 patients without complications. In one patient with previous sternotomy, LAAO was not feasible. Blood product transfusion was necessary in one patient, due to significant chest wall bleeding requiring thoracoscopic re-exploration. No stroke or thromboembolic events were observed. Left hemidiaphragm paralysis requiring plication occurred in 3 patients (1.5%). Hospital mortality was 0%. Mean LOS time was 2.2 days (range 1–10 days) and all patients were discharged home. Imaging follow-up was complete in 157 cases (81%) and was achieved by TEE in 91 patients while the remaining 66 underwent cardiac CT scan. Two patients had residual flow in the left atrial appendage and oral anticoagulant was continued at follow-up.

**CONCLUSIONS:**

Robotic-assisted LAAO is safe with satisfactory outcome both in an isolated setting and with concomitant hybrid ablating procedures for patients with AF.

## GRAPHICAL ABSTRACT

**Figure:**
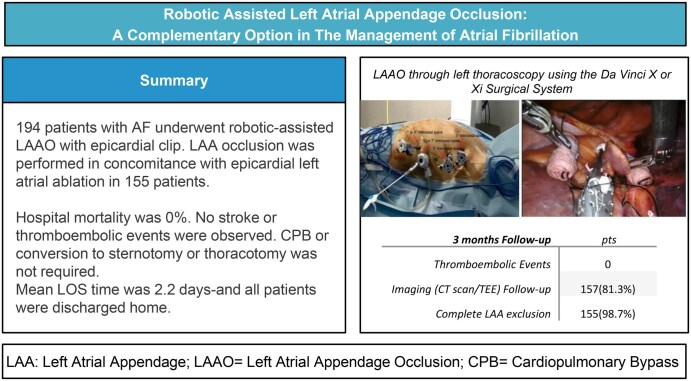

## INTRODUCTION

Atrial fibrillation (AF) is the most prevalent arrhythmia worldwide and carries a 3- to 5-fold risk of thromboembolic stroke (TES) [1, 2].

Oral anticoagulants (OACs) are considered the treatment of choice to prevent TES in these patients, with a documented relative incidence decreasing by 50–80% [3]. OACs, however, are associated with an increased risk of bleeding, especially in elders, and about 10% of patients present a relative or absolute contraindication [4], while a significative number of them spontaneously discontinue OAC in 1–2 years.

Mechanical left atrial appendage (LAA) exclusion, by either endocardial or epicardial approach, has proven to be a viable and safer alternative, demonstrating a reduction of TES incidence comparable to OAC [5, 6]. Although prospective randomized trials such as PROTECT AF and PREVAIL [5, 7] have shown the safety and efficacy of a transcatheter LAA occlusive device (Watchman, Boston Scientific, St Paul, MN) in preventing stroke in AF compared to Warfarin, anatomical features, previous surgical or percutaneous atrial septum repair, intolerance to OAC or dual antiplatelet therapy, may represent a relative contraindication to the procedure.

Surgical left atrial appendage occlusion (LAAO) in patients with AF is currently recommended in patients undergoing cardiac surgery. The LAAOS III trial [8] has shown that in patients with AF and a CHA_2_DS_2_-VASc score of at least two patients who undergo cardiac surgery for another indication, the risk of stroke or systemic embolism was lower when concomitant LAAO was performed.

Increasing evidence demonstrating the clinical benefits of LAAO and the development of surgical minimally invasive and thoracoscopic techniques have further favoured both stand-alone LAAO and its combination with hybrid ablation procedures.

Epicardial atrial clips have proven to be a valuable tool, adapting to the thoracoscopic approach, with excellent results in terms of safe and durable LAA occlusion [9, 10].

The use of robotic technology with its advantages in terms of magnified visualization and enhanced dexterity, can facilitate such procedure potentially improving clinical outcomes, minimizing surgical trauma and perioperative complications.

The aim of this observational, retrospective, multicentre study is to demonstrate safety, feasibility, and efficacy of LAAO performed by means of a robotic-assisted approach.

## MATERIALS AND METHODS

### Study design, patients and technology

This is a multicentre, retrospective study of adult patients with known history of AF that underwent LAAO with a robotic-assisted approach.

Data were collected in each institution’s database and retrospectively reviewed with the goal to assess safety and efficacy of the procedure.

Indications for AF epicardial ablation and LAAO followed Heart Rhythm Society/European Heart Rhythm Association/European Cardiac Arrhythmia Society guidelines [11]. Isolated LAA clip closure was reserved for patients with contraindication to OACs due to haemorrhagic risk, poor adherence to therapy or contraindication to any percutaneous device. Exclusion criteria include LAA thrombus identified through perioperative transesophageal echocardiography (TEE).

Robotic procedures were performed with Da Vinci X or Xi Surgical System (Intuitive Surgical Inc., Sunnyvale, CA). The implanted epicardial clips were either AtriClip PRO-2 or PRO-V (AtriCure Inc, Mason, OH). Both designs are suited for thoracoscopic procedure.

All procedures were TEE guided with imaging performed by the same TEE-credentialed cardiac anaesthesiology team in each institution.

A failed epicardial LAA exclusion was defined as an appendage with either a stump >10 mm or residual flow (detected by TEE) or contrast enhancement by cardiac CT scan (computed tomography angiography [CTA]) at follow-up, as previously described [12].

Given the observational and retrospective nature of the study, the need for informed consent was waived.

### Preoperative diagnostic workup and data collection

Preoperative evaluation was completed for all patients according to the protocol of the reference centre.

Specifically, cardiac CTA or magnetic resonance imaging (MRI) were routinely performed to delineate the complex relationships between the left atrium, pulmonary veins, and surrounding structures. Imaging was also useful to rule out LAA thrombus.

Coronary angiography was usually not performed, unless patients presented risk factors or family history of ischaemic heart disease. In addition, spirometry was considered for patients with chronic obstructive pulmonary disease (COPD).

Follow-up data were obtained during outpatient follow-up visits and phone interviews, while occlusion of LAA was verified with either TEE or cardiac CTA approximately 3 months after the procedure.

### Surgical technique

The robotic-assisted approach for LAAO was performed as previously described [13, 14] and was conducted similarly for both stand-alone LAAO and LAAO combined with epicardial AF ablation. Briefly, the procedure consists of a unilateral, left-sided approach under general anaesthesia. After left lung deflation four robotic ports, used, respectively, for a 30° high-definition robotic camera, for bipolar dissector and robotic forceps, are inserted in ‘hockey-stick’ fashion and robotic arms are subsequently docked.

**Figure 1:**
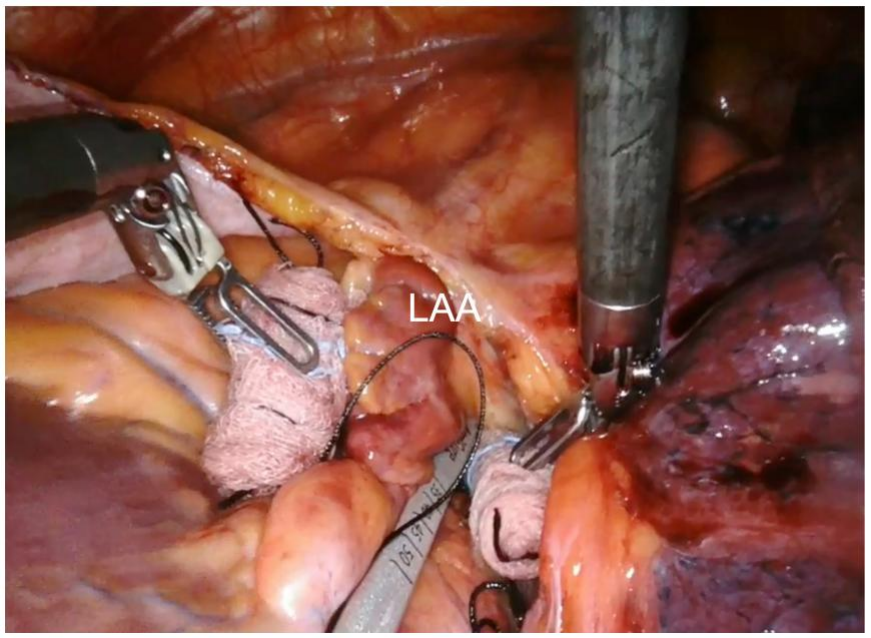
Left atrial appendage (LAA) sizing

**Figure 2:**
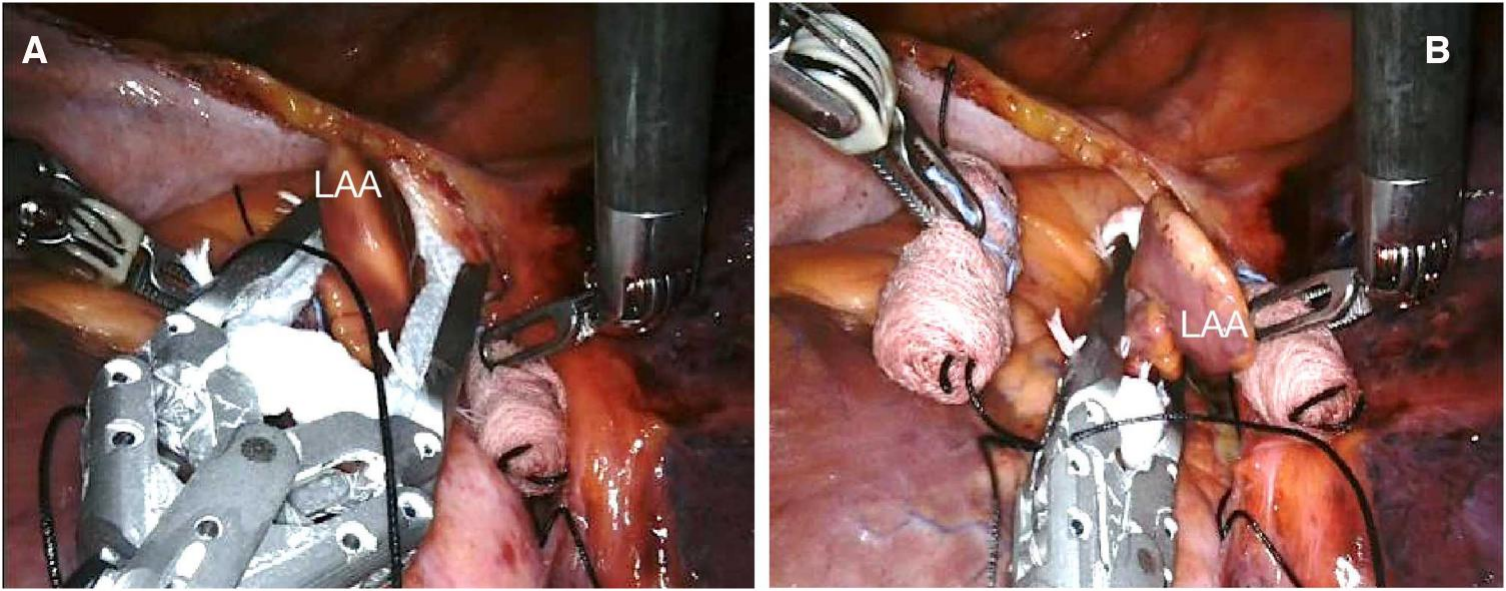
Placing AtriClip at the base of the LAA (**A**) and closing the device (**B**)

**Figure 3:**
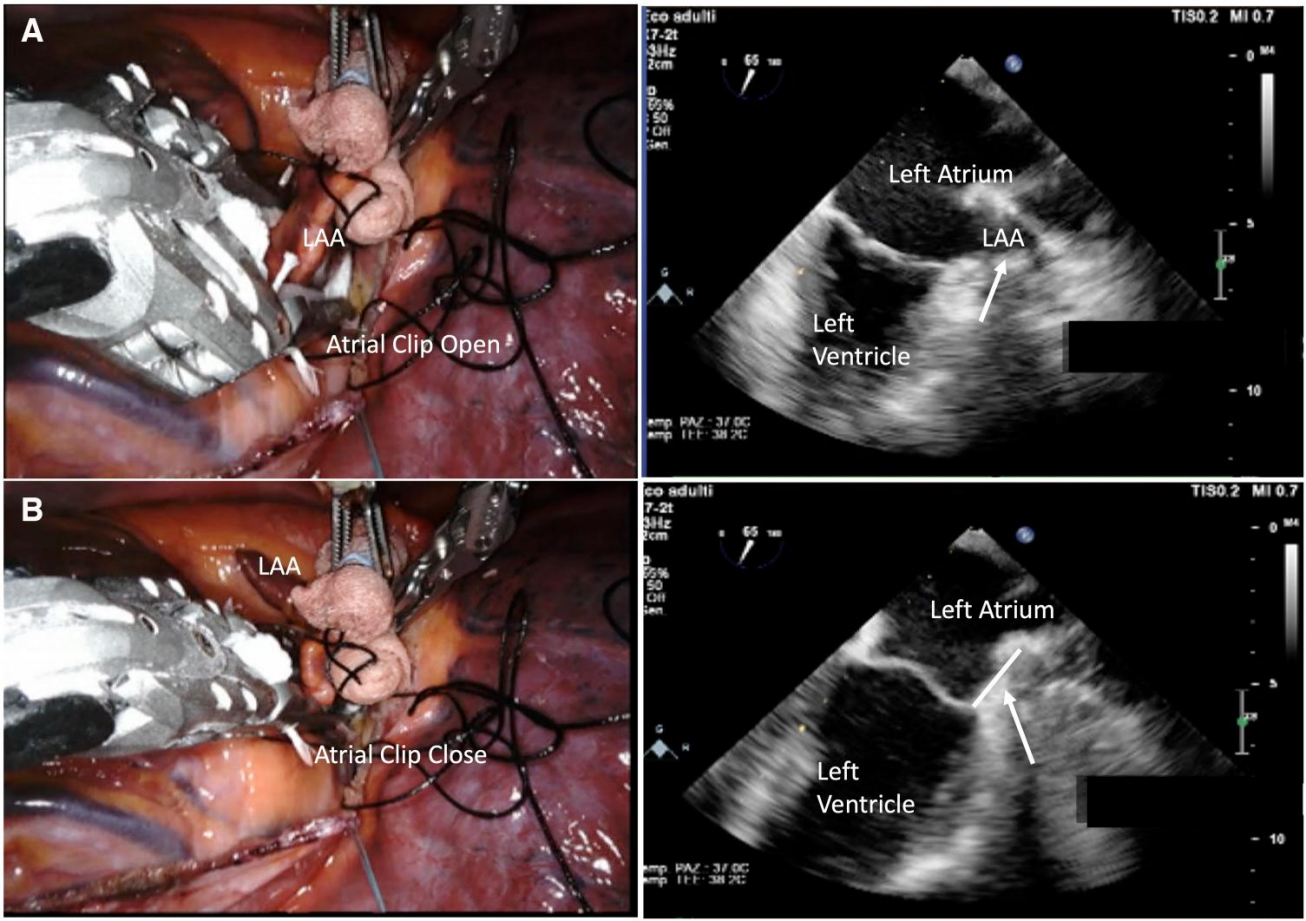
TEE monitoring before (**A**) and after (**B**) Atriclip release without significant residual stump

**Figure 4:**
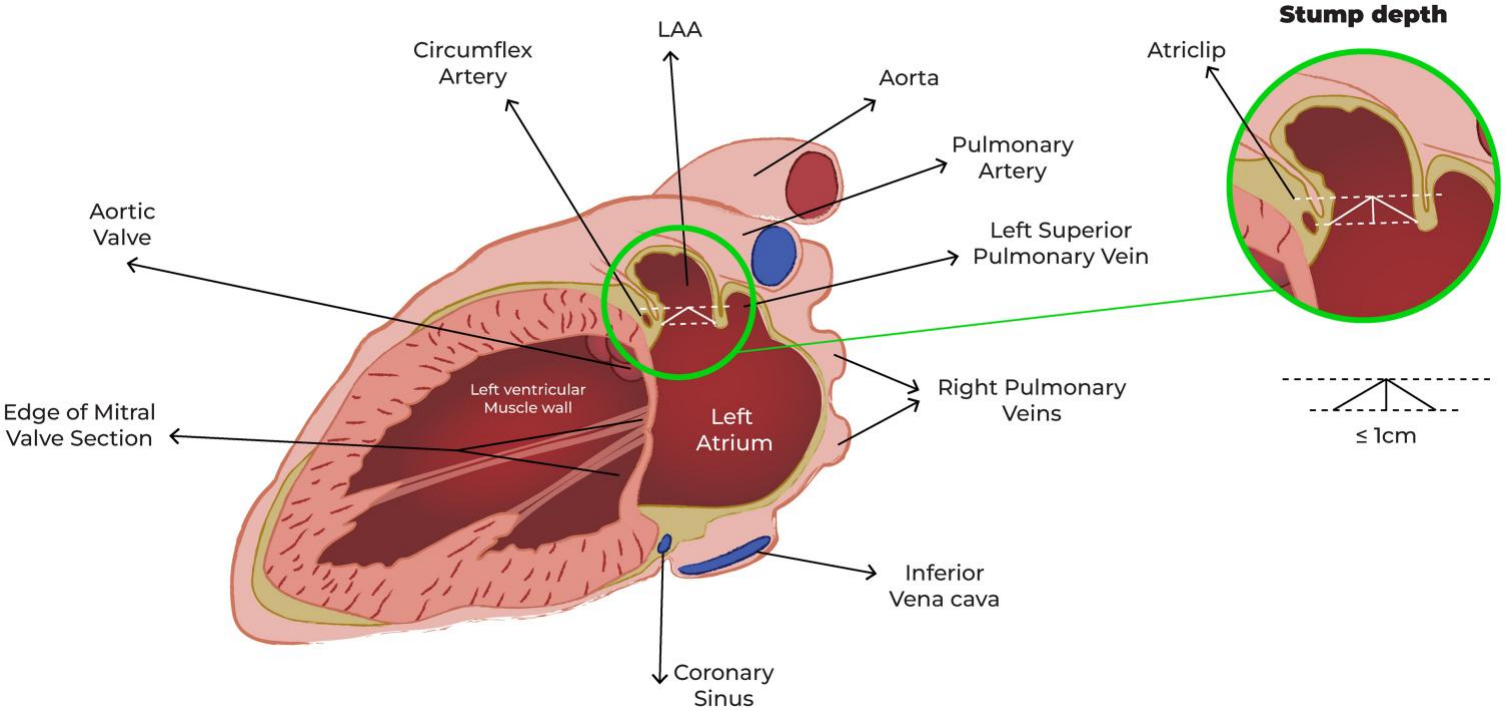
Diagram of echocardiographic measurement of residual stump after Atriclip release

The pericardium is longitudinally opened under the phrenic nerve and the LAA is identified. In patients undergoing concomitant left atrial epicardial ablation, at first, the surgeon completes the designated lesion set using a 3 cm, self-irrigated, radiofrequency EP-Sense probe (Atricure Inc., Mason, OH). Then, the LAA is sized (Fig. 1) and the epicardial clip is placed (Fig. 2A and B). The proper clip positioning is carefully guided by TEE and repeated as needed until proper closure is achieved (Fig. 3A and B). Therefore, prior to releasing the clip, confirmation of no residual flow on colour Doppler and sizing of the residual LAA stump are critical. The latter is measured as the height of a triangle where the base is represented by an ideal line between the left circumflex artery and the reference counterpoint on the lateral ridge, and its apex being the deepest point of the stump (Fig. 4) Continuous electrocardiographic monitoring is performed during clip placement to rule out ST-segment changes due to accidental impingement of the circumflex artery.

Of note, in the first 36 patients undergoing epicardial AF ablation and LAAO, we have combined unilateral robotic access with subxiphoid pericardioscopic access. Due to perceived suboptimal visualization of the left atrial posterior wall for the purpose of its ablation, as well as the occurrence of a subxiphoid incisional hernia in two patients, we have modified this approach into a total port-access robotic approach.

### Statistical analysis

Continuous variables were expressed as mean with standard deviation, whereas categorical variables were calculated as percentages of absolute numbers, using a standard Excel (Microsoft 365; Microsoft Corp) statistical package.

## Human Subjects

This study adheres to all pertinent national regulations and was conducted in accordance with the principles outlined in the Helsinki Declaration. Local Institutional Review Board approval was obtained (PRO00039130, approved 30 December 2020 by the Medical College of Wisconsin Institutional Review Board; Prot. 46/24 GAV approved 22 October 2024 by the ethics committee of IRCCS Humanitas Clinical Institute Rozzano).

## RESULTS

### Study population

Between August 2019 and June 2024, 194 patients underwent LAAO through left thoracoscopy using the Da Vinci X or Xi Surgical System (166 patients at the Medical College of Wisconsin, Milwaukee and 28 at the Humanitas Gavazzeni-Castelli, Bergamo). Within this cohort, 155 (80%) underwent concomitant epicardial ablation as part of a hybrid AF treatment. Baseline demographics are described in Table 1.

**Table 1:** Preoperative characteristics

	Patient number: 193
Mean Age (years)	67.7 ± 10.1
Gender, male	136 (70.4%)
Mean BMI (kg/m^2^)	31.8 ± 6.5
Mean CHA_2_DS2 VASc score	3.0 ± 1.6
Mean HAS-BLED score	2.7 ± 1.3
Previous cardiac surgery	14 (7.2%)
Contraindication to OACs	25 (12.9%)
Class I/III antiarrhythmic drug	145 (75%)
Mean EF, %	54.4% ± 10.4%
Type of AF	
PAF	21 (11%)
PsAF	118 (61%)
LSAF	55 (28%)
Mean AF duration (months)	72 ± 48
Previous CVE	*127 (66%)*
Previous transcatheter LAA exclusion	*1 (0.5%)*
Medical history	
Hypertension	*148 (77%)*
Diabetes	*54 (28%)*
COPD	*12 (6.2%)*
Dyslipidaemia	*96 (50%)*
Stroke	*7 (3.6%)*
Thyroid disorders	*51 (26.4%)*
Chronic kidney disease	*7 (3.6%)*
Obstructive sleep apnea	*29 (15%)*

BMI: body mass index; OACs: oral anticoagulants; EF: ejection fraction; AF: atrial fibrillation; PAF: paroxysmal AF; PsAF: persistent AF; LSAF: long-standing AF; COPB: chronic obstructive pulmonary disease.

Notably, 12.9% of patients had a relative or absolute contraindication to OAC due to history of major bleeding. In one case, due to known history of nickel allergy, a nickel-free epicardial clip device (AtriClip Pro-V) was utilized. One patient had a previously failed attempt to percutaneous LAA closure with a Watchman device.

Fourteen patients (7.2%) had history of prior cardiothoracic procedures, such as pericardial window, CABG (coronary artery bypass graft), mitral valve repair/replacement, aortic valve replacement, ventricular septal myectomy and atrial septal repair.

### Intra- and postoperative results

Complete LAAO was successfully achieved in 193 (99.5%) patients. In one patient not eligible to percutaneous approach and previously undergone mitral valve repair the procedure was aborted due to dense intrapericardial adhesions.

In other patients with prior sternotomy, pericardial adhesions were identified and managed successfully with the robotic approach. Intra- and postoperative characteristics are depicted in Table 2. Importantly, none of the patients required conversion to full sternotomy, thoracotomy or cardiopulmonary bypass (CPB) support. Blood product transfusion was necessary in one patient with coagulopathy and chest wall bleeding requiring thoracoscopic revision on postoperative day 2.

**Table 2:** Intraoperative and postoperative characteristics

	Patient number: 193
Epicardial AF ablation + LAA occlusion	155 (80%)
LAA occlusion	39 (20%)
Successfully procedure complete	193 (99.4%)
Concomitant surgical procedure	14 (7.2%)
Pericardial window	1 (0.5%)
Coronary artery bypass graft	7 (3.6%)
Mitral valve repair/replacement	3 (1.6%)
Aortic valve replacement	1 (0.5%)
Ventricular septal myectomy	1 (0.5%)
Atrial septal repair	1 (0.5%)
LAA clip size	
35	15 patients
40	24 patients
45	53 patients
50	109 patients
CPB required	0
Conversion to full sternotomy or mini-thoracotomy	0
Operative-room extubation	186 (96%)
Blood product transfusion	1 (0.5%)
Mean mechanical ventilation time (hours)	3 ± 1
Mean ICU time (hours)	16 ± 3
Mean LOS (days)	2.2 (range 1–10)

LAA: left atrial appendage; CPB: cardiopulmonary bypass; ICU: intensive care unit; LOS: length of stay.

Atriclip PRO2 was implanted in 182, while Atriclip Pro-V in 19 patients. In eight patients, a second clip was needed due to incomplete LAAO following release of the first one.

Operative room extubation was achieved in 96% of patients, admitted to a regular surgical floor or sub-intensive unit according to each individual institutional policy. In eight patients (4%), temporary intensive care unit (ICU) admission became necessary because of either persistent hypoxia secondary to known COPD or due to the need for weaning inotropic support. ICU length stay was less than 24 h.

There was no operative mortality. No cerebral or peripheral thromboembolic events were observed. Four patients (2%) in the first phase of our experience experienced postoperative left hemidiaphragm paralysis requiring surgical plication in three cases, while spontaneous resolution was observed in the remaining one.

Lastly, four patients (11.1%) among those undergoing robotic-assisted hybrid ablation with a subxiphoid access developed a subxiphoid incisional hernia which required surgical repair. Postoperative complications are summarized in Table 3.

**Table 3:** Postoperative complications

	Patient number: 193
Hospital mortality	0
Thromboembolic events	0
Bleeding requiring thoracoscopic revision	1 (0.5%)
Thoracentesis for new pleural effusions	5 (3%)
Left hemidiaphragm paralysis	4 (2%)
Subxiphoid incisional hernia	4 (2%)

The median duration of hospitalization was 2.2 days (range 1–10 days). During this period, antiarrhythmic medications were reintroduced while OACs were prescribed only in patients without contraindications. All patients were discharged home.

### Follow-up

Follow-up consisted of an initial outpatient visit and/or phone interviews at 3 months. Patients were subsequently scheduled for imaging follow-up (CTA or TEE) to confirm proper LAAO. To date, neither deaths nor thromboembolic complications have occurred during follow-up.

Imaging follow-up was complete in 157 cases (81%): 91 patients underwent TEE while 66 patients cardiac CT scan. Two patients (1.2%) had residual flow in LAA, as a sign of incomplete occlusion. For both patients, OAC was continued at follow-up.

## DISCUSSION

To the best of our knowledge, this multicentre experience with robotic-assisted LAAO in patients diagnosed with AF, as an either isolated procedure or combined with epicardial left atrial ablation, represents the largest one reported to date. AF is an important risk factor for TES and different studies report 90% of atrial thrombi within the LAA [15]. In AF, the LAA undergoes negative remodelling due to increased atrial pressure and volume, which not only leads to decreased compliance but also decreased Doppler velocities increasing the risk of thrombus formation [4]. Anticoagulation is the mainstay of stroke prevention in this population [16]. Despite its well-established benefits, however, it is limited by either major bleeding or poor patient’s adherence.

LAAO represents an alternative strategy to reduce the risk of stroke. Current European guidelines [17] recommend surgical LAA occlusion in patients with AF undergoing cardiac surgery (Class IB).

With the advancement of technology and the advent of thoracoscopic epicardial ablation of AF, surgeons have an invaluable opportunity to manage the LAA, exploiting the benefits of minimally invasive treatment.

Observational data showed that LAA closure using an epicardial clip is a feasible and safe procedure and should be considered as an adjunct to oral anticoagulation in patients with AF undergoing endoscopic or hybrid AF ablation (class IIA), while stand-alone endoscopic surgical closure may be considered in patients with AF and contraindications for long-term anticoagulant (class IIB).

In this setting, the advent of robotic technology in cardiac surgery has brought a new impetus in the development of the procedure, exploiting the important advantages of robotic instruments described in several papers such as magnified visualization, manoeuvrability and precision [18, 19] and minimizing surgical trauma on wall chest.

We recognize that a robotic approach in the management of the LAA certainly requires, at the minimum, advanced robotic skills within the realm of cardiac and thoracic surgery. Both our centres have extensive experience in robotic procedures. Having trained several other colleagues both in the USA and Europe, we do believe that a minimum of 20 proctored cases for groups with prior robotic experience is necessary. Given its novelty, there are no established standards or requirements associated with individual hospital’s credentialing.

Considering the growing evidence on the effectiveness and safety of the epicardial linear LAA closure device Atriclip [20, 21], we chose this device in the two iterations PRO2 and PRO-V for our procedures. An important goal is the flexibility of the distal end that adapts to different angles and the ability to be used within a 12-mm port. While some LAA anatomical shapes might limit the use of endocardial devices, Atriclip has no anatomical restrictions and we have used it for every type and size of LAA (Cauliflower, Chicken Wing, Windsock, Cactus) without compromising the quality of exclusion.

In our experience, such approach has demonstrated to be safe, even in more complex cases such as patients with prior sternotomy, as demonstrated by the absence of intraoperative mortality, need for CPB support and conversion to sternotomy or thoracotomy due to injury or major bleeding, as also described in the study of Antaki *et al.* [22].

After an initial learning period [23], anatomical characteristics of the patients such as body size and habitus were not a limitation for the robotic approach. Preoperative evaluation remains paramount, particularly in the early stage of the learning curve to tailor such procedure to each individual patient’s risk factors and specific anatomy.

In addition to lack of operative mortality and major cardiovascular events, we also observed a low rate of minor complications. These were mostly represented by wound complications, such as Incisional subxiphoid hernias which occurred in very obese patients. These occurrences inspired the transition towards a total robotic approach for patients undergoing concomitant ablation as part of their hybrid AF treatment [14].

In the early stage of the experience, 2% of patients had left hemidiaphragm paralysis requiring plication, while there were no cases in the later stages due to improved dexterity and safer entrance of the pericardial cavity yet reiterating the importance of a learning curve.

The decreased invasiveness of the robotic approach brings additional advantages as far as postoperative care. The vast majority of patients (96%) were extubated in the operating room, did not require admission to the ICU and blood products transfusion and were mobilized within the first few hours of the procedure.

Postoperative pain was well controlled and did not affect patient recovery. All patients were discharged home after a short hospitalization, with rapid functional recovery and resumption of daily life activities.

Concerns may be raised about the cost-effectiveness of the procedure. Robotic surgery is significantly more expensive than other established approaches for similar clinical indications. To truly be considered effective, in addition to potentially decrease complications, such approach has to be able to cut overhead costs in other critical aspects of patient’s management, as described in our previous study [24]. In our experience, a robotic-assisted approach to the LAA has dramatically impacted length of stay (LOS; median 2.2 days), need for highly specialized care (i.e. ICU admission), blood product transfusion and pain management.

In addition to safety, our study demonstrated the effectiveness of the procedure both intraoperatively and at follow-up. As recommended in several studies [20, 12, 25], residual LAA stump after epicardial LAAO should be less than 10 mm. Longer stumps have been associated with increased risk of left atrial thrombosis and TES [26]. This remarks the importance of TEE guidance during the procedure to achieve optimal position of the clip prior to its final release, eventually allowing its repositioning or the use of additional clips if the initial morphology of the LAA is challenging.

The success of the procedure was confirmed also at follow-up where cardiac imaging (CTA or TEE) plays a key role, also identifying the patients with incomplete closure and enabling them to be treated.

Finally, the LAA is a known arrhythmogenic site and represents a site of non-pulmonary vein triggers in almost 25% of patients with persistent AF [27]. One possible explanation involves atrial remodelling caused by AF that results in ultrastructural cellular changes with atrial electrical/mechanical functional abnormalities. These changes may promote arrhythmogenicity via a combination of enhanced automaticity, triggered activity, and localized micro-reentry. Di Biase *et al.* studied 987 patients who underwent repeat catheter ablation for AF. The study concluded that 27% of the patients had aberrant firing originating from the LAA, and in 8.7% the LAA was the only source of AF [28]. This was likely secondary to LAA having autonomic innervation which predisposes it to have arrhythmogenic qualities. Friedman *et al.* conducted a large meta-analysis which showed that LAA electrical isolation resulted in a significant decrease in AF and atrial tachycardia [29]. Epicardial occlusion of the LAA with a clip induces tissue infarction and transmural necrosis, resulting in decreased voltage transmission and complete electrical isolation. Due to less substrate availability, AF is less likely to occur, increasing the success of AF ablation procedures as well.

In conclusion, the benefits of LAAO are numerous and include not only the reduction of thromboembolic risk but also positive impact on the success of ablation techniques for the treatment of AF and on arrhythmia control.

### Limitations

Our study presents several limitations. The first being represented by its retrospective nature with expected patient selection and observer biases that are inherent to the introduction of a newer technique. The results are also the cumulation of the experience of two centres that started robotic-assisted AF surgery at different times. The team at Humanitas Gavazzeni started the procedure about 3 years later and this may have influenced some aspects of the initial postoperative management, such as operative room extubation and duration of the LOS. Inherent differences between patient populations at the two centres, including patient characteristics (e.g. body mass index), must be considered, although different sample sizes between the two centres may have mitigated such bias. Lastly, generalizations are difficult to make based on individual practices, level of experience and availability of certain technological features. Our institutions routinely manage a high volume of robotically assisted cardiothoracic surgery cases, and the authors are surgeons with advanced robotic skills, which may not reflect the general practice. Larger cohorts, longer follow-up and, possibly, randomized studies including comparable control groups, such as patients on OACs only or patients treated with endocardial approach, will be necessary to have a causal conclusion of efficacy.

## CONCLUSIONS

Robotic LAA epicardial occlusion has proven safe with satisfactory outcome in this population. Decreased invasiveness combined with improved structural visualization limits operative trauma with minimal morbidity, shorter hospitalization and faster functional recovery, adding to the armamentarium of available options, particularly for patients with contraindication to OAC and percutaneous closure.

## Data Availability

The data underlying this article will be shared on reasonable request to the corresponding author.

## ABBREVIATIONS

AF: Atrial fibrillation
BMI: Body mass index
CTA: Computed tomography angiography
COPD: Chronic obstructive pulmonary disease
ICU: Intensive care unit
LAA: Left atrial appendage
LAAO: Left atrial appendage occlusion
LOS: Length of stay
LSAF: Long-standing atrial fibrillation
OAC: Oral anticoagulant
PAF: Paroxysmal atrial fibrillation
PsAF: Persistent atrial fibrillation
TEE: Transesophageal echocardiography
TES: Thromboembolic stroke
